# Sequence-Based Prediction for Vaccine Strain Selection and Identification of Antigenic Variability in Foot-and-Mouth Disease Virus

**DOI:** 10.1371/journal.pcbi.1001027

**Published:** 2010-12-09

**Authors:** Richard Reeve, Belinda Blignaut, Jan J. Esterhuysen, Pamela Opperman, Louise Matthews, Elizabeth E. Fry, Tjaart A. P. de Beer, Jacques Theron, Elizabeth Rieder, Wilna Vosloo, Hester G. O'Neill, Daniel T. Haydon, Francois F. Maree

**Affiliations:** 1 Boyd Orr Centre for Population and Ecosystem Health, University of Glasgow, Glasgow, United Kingdom; 2 Institute of Biodiversity, Animal Health and Comparative Medicine, College of Medical, Veterinary and Life Sciences, University of Glasgow, Glasgow, United Kingdom; 3 Transboundary Animal Diseases Programme, Onderstepoort Veterinary Institute, Agricultural Research Council, Onderstepoort, South Africa; 4 Department of Microbiology and Plant Pathology, University of Pretoria, Pretoria, South Africa; 5 Division of Structural Biology, The Henry Wellcome Building for Genomic Medicine, Headington, United Kingdom; 6 Bioinformatics and Computational Biology Unit, University of Pretoria, Pretoria, South Africa; 7 Foreign Animal Disease Research Unit, United States Department of Agriculture, Agricultural Research Service, Plum Island Animal Disease Center, Greenport, New York, United States of America; 8 Biochemistry Division, North-West University, Potchefstroom, South Africa; University of New South Wales, Australia

## Abstract

Identifying when past exposure to an infectious disease will protect against newly emerging strains is central to understanding the spread and the severity of epidemics, but the prediction of viral cross-protection remains an important unsolved problem. For foot-and-mouth disease virus (FMDV) research in particular, improved methods for predicting this cross-protection are critical for predicting the severity of outbreaks within endemic settings where multiple serotypes and subtypes commonly co-circulate, as well as for deciding whether appropriate vaccine(s) exist and how much they could mitigate the effects of any outbreak. To identify antigenic relationships and their predictors, we used linear mixed effects models to account for variation in pairwise cross-neutralization titres using only viral sequences and structural data. We identified those substitutions in surface-exposed structural proteins that are correlates of loss of cross-reactivity. These allowed prediction of both the best vaccine match for any single virus and the breadth of coverage of new vaccine candidates from their capsid sequences as effectively as or better than serology. Sub-sequences chosen by the model-building process all contained sites that are known epitopes on other serotypes. Furthermore, for the SAT1 serotype, for which epitopes have never previously been identified, we provide strong evidence – by controlling for phylogenetic structure – for the presence of three epitopes across a panel of viruses and quantify the relative significance of some individual residues in determining cross-neutralization. Identifying and quantifying the importance of sites that predict viral strain cross-reactivity not just for single viruses but across entire serotypes can help in the design of vaccines with better targeting and broader coverage. These techniques can be generalized to any infectious agents where cross-reactivity assays have been carried out. As the parameterization uses pre-existing datasets, this approach quickly and cheaply increases both our understanding of antigenic relationships and our power to control disease.

## Author Summary

New strains of viruses arise continually. Consequently, predicting when past exposure to closely related strains will protect against infection by novel strains is central to understanding the dynamics of a broad range of the world's most important infectious diseases. While previous research has developed valuable tools for describing the observed antigenic landscapes, our ability to predict cross-protection between different viral strains depends almost entirely on cumbersome and expensive live animal work, often restricted to model species rather than the natural host. The development of computer-based approaches to the estimation of cross-protection from viral sequence data would be hugely valuable, and our study represents a significant step towards this research goal.

## Introduction

The genetically highly variable nature of RNA viruses [1] has been extensively documented in pathogens such as foot-and-mouth disease virus (FMDV) and influenza virus. A direct consequence of this phenomenon is that inactivated or attenuated vaccines derived from some such highly variable viruses confer protection only against closely related field strains [2], as has been amply demonstrated during the 2009 influenza A (H1N1) pandemic [3]. This feature of the viruses makes it particularly important to estimate the cross-reactivity, and therefore the likely cross-protection, between sera derived from the vaccine strain and field viruses [4], [5].

The emergence of antigenically novel viruses, against which existing vaccines do not provide adequate protection, may require the selection of new vaccine seed strains. Currently, where no appropriate vaccine exists, field isolates are, when possible, adapted for vaccine production, amplified and then processed into vaccines [6]. Only at this stage can the new vaccines be inoculated into animals and tested for efficacy *in vitro* and subsequently *in vivo*. Due to the time and expense required, there is a limit to the number of isolates that can be submitted to undergo this procedure, and a sub-optimal choice of vaccine strain may therefore be made. An *in silico* predictor that identifies those strains likely to provide the broadest cross-protection could therefore substantially enhance capacity to develop appropriate vaccines rapidly and effectively, whilst minimising the cost and the need for animal experimentation.

FMDV is ideal for such an approach to vaccine strain selection. It is a positive-sense, single-stranded RNA virus, the prototype member of the genus *Apthovirus* of the family *Picornaviridae*. It exhibits great genomic variability, with 32–33% and 53% amino acid variability in our data, within and between serotypes respectively, in the immunogenically important structural proteins, VP1-VP3 (similar variability has previously been observed in VP1 across all serotypes [7]). Its seven serotypes are not cross-reactive, but individual vaccines can often protect against large groups of genetically diverse viruses within a serotype. Nevertheless there are also antigenically distinct subtypes within each serotype, and this should allow the discrimination of antigenically important changes from other substitutions. The virus is endemic in sub-Saharan Africa where six of the seven serotypes occur, and the South African Territories (SAT) types 1, 2 and 3 display appreciably greater intratypic genomic variation than the traditional “Euro-Asian” types [8]–[13]. Indeed, distinct genetic variants exist within these serotypes, with the serotypes being divided into topotypes based on genetic differences [7].

The variability of all SAT FMDV serotypes requires both a range of vaccines to provide protection within serotypes and accurate cross-reactivity testing to guide vaccine selection, with implications for the control of the disease by vaccination if either of these is not available. Despite similar genomic variability, SAT2 exhibits significantly higher intratypic antigenic variability than SAT1 [11]. Such a pair of serotypes with similar genetic but different antigenic characteristics provide an excellent testing ground for studies of the genetic basis of antigenic variability. Furthermore, SAT2 viruses are the causative agent in most outbreaks of FMDV in cattle in sub-Saharan Africa, and SAT1 is also widely dispersed, though mostly maintained through persistent infections of African buffalo. This makes them the most important serotypes to study in the region.

The outer capsid proteins – VP1, VP2 and VP3 – are directly involved in antigenicity and a large proportion of residues are exposed on the virion surface (40% in the structure used in this paper). Amongst the exposed residues are epitopes recognised by the host immune system. All serotypes are believed to share the major antigenic site on the flexible G-H loop of the VP1 protein, which is highly variable between even closely related strains. This is the only site to have been identified with monoclonal antibody (MAb) escape mutants for a single SAT2 virus [14], and none have been for SAT1. However, this and at least four additional sites have been implicated as neutralising epitopes for serotype O [15]–[17], and further epitopes have been mapped for viruses from serotypes A, O and C using MAbs [15]–[24].

This antigenic variability is reflected in the virus neutralisation (VN) titres [25], which provide an *in vitro* measure of whether the sites that contribute to the neutralization of the virus remain sufficiently similar to cross-react. Virus neutralisation is not the only important determinant of protection [26]; nevertheless the VN test (VNT) is one of the standard tests for cross-reactivity and it is considered to provide the most definitive serological results [6]. Specifically, the current approach uses VNTs to quantify antigenic relationships through “r_1_-values” – the ratio of the heterologous to homologous titres, with a ratio close to 1 indicating the viruses are antigenically similar. Generally r_1_-values in the range of 0.4–1.0 are considered to be indicative of reasonable levels of cross-protection, whilst all values being below 0.2 for a given isolate indicate the need for new vaccine strain development [27], with 0.3 also proposed as a single threshold [28]. Many sources of variation are known to influence the neutralisation titres. However, standard approaches to obtaining r_1_-values do not fully account for these different sources of variability [29]. In order to maximise information available from neutralisation tests we developed a simple statistical methodology using multiple data sources, combining data from multiple experiments conducted at different times.

The availability of sequence data and related titres from VN testing provides the opportunity to directly relate cross-reactivity to sequence variation. This relationship would allow prediction of an important component of vaccine efficacy for candidate vaccine seed strains, and rapid identification of vaccine match without the need for new serology work for existing vaccines.

The aim of the current study is to develop an *in silico* tool to predict vaccine efficacy using sequence data, neutralising titres and structural information, and use this tool to identify and quantify the significance of epitopes of the viruses. We have obtained a broad spectrum of SAT1 and SAT2 isolates which were sequenced, and have generated sera from representative viruses. An extensive serological dataset was generated from VNTs. We have also used a novel, and currently the only, crystallographic structure for any SAT serotype to identify surface-exposed residues on the capsid. Specific objectives were to (i) generate improved statistical methods of estimating r_1_-values that maximise the efficient use of available experimental data, (ii) relate these estimated antigenic differences to sequence variation, (iii) use this relationship to predict vaccine match for viruses from sequence information, and to predict neutralisation titres, cross-reactivity and hence coverage for vaccine strains, and then (iv) to identify areas of the capsid containing epitopes.

## Results

### Virus isolates, sequencing and VNTs

Twenty SAT1 and twenty-two SAT2 viruses (Table 1) representative of different topotypes were selected, and full capsid sequences were generated where not already available. This collection constitutes fully two thirds of all isolates for which full capsid sequences exist. Cattle sera were prepared against three SAT1 and four SAT2 strains. VNTs were carried out with 138 different pairs of protective strain and challenge virus, 59 of the 60 possible SAT1-SAT1 pairs and 83 of the 88 SAT2-SAT2, with between 1 and 11 repeats of each, giving a total of 246 SAT1 and 320 SAT2 titres. This included replicates within individual experiments, and repeats with different sera and in different batches (see Materials and Methods for an explanation of the terminology), in order to determine the significant sources of variability.

**Table 1:** FMDV isolates used in this study.

Serotype	Virus strain	Topotype	Passage history	Country of origin	GenBank Accession No.
SAT1	KNP/196/91*	1	PK1RS1	South Africa	AF283429
SAT1	KNP/148/91	1	PK1 RS5	South Africa	GU194495
SAT1	ZIM/HV/3/90	1	BTY1 RS3	Zimbabwe	GU194496
SAT1	ZIM/GN/13/90	1	BTY1 PK1 RS3	Zimbabwe	GU194497
SAT1	KNP/41/95	1	PK1 RS4	South Africa	GU194498
SAT1	SAR/9/81*	1	Epithelium	South Africa	DQ009715
SAT1	NAM/307/98	2	PK1 RS4	Namibia	AY770519
SAT1	ZIM/6/94	2	PK1 RS3	Zimbabwe	GU194500
SAT1	TAN/37/99	3	BTY1 RS4	Tanzania	DQ009718
SAT1	ZAM/2/93	3	PK1 RS3	Zambia	DQ009719
SAT1	ZIM/25/90	3	BTY2 RS4	Zimbabwe	GU194499
SAT1	MOZ/3/02	3	PK1 RS5	Mozambique	DQ009720
SAT1	KEN/5/98	3	BTY1 RS3	Kenya	DQ009721
SAT1	UGA/3/99	4	BTY1 RS4	Uganda	DQ009722
SAT1	UGA/1/97	5	PK1 RS4	Uganda	AY043300
SAT1	NIG/5/81*	7	BTY2 RS2	Nigeria	DQ009723
SAT1	SUD/3/76	7	BTY1 RS3	Sudan	DQ009725
SAT1	NIG/15/75	8	BTY1 RS3	Nigeria	DQ009724
SAT1	NIG/8/76	8	BTY1 RS5	Nigeria	GU194503
SAT1	NIG/6/76	8	BTY1 RS5	Nigeria	GU194502
SAT2	KNP/19/89*	I	BHK4	South Africa	DQ009735
SAT2	KNP/2/89	I	CFK2 RS2 BHK4	South Africa	GU194488
SAT2	KNP/51/93	I	PK1 RS6	South Africa	GU194489
SAT2	ZIM/1/88	I	CFK1 RS4	Zimbabwe	GU194491
SAT2	SAR/16/83	I	B1 BHK8	South Africa	DQ009734
SAT2	ZIM/14/90	II	BTY1 RS3	Zimbabwe	DQ009728
SAT2	ZIM/17/91	II	BTY2 RS4	Zimbabwe	DQ009727
SAT2	ZIM/GN/10/91	II	BTY2 PK1 RS3	Zimbabwe	GU194493
SAT2	RHO/1/48	II	BTY2 RS2	Zambia	AJ251475
SAT2	ZIM/7/83*	II	B1 BHK5 B1	Zimbabwe	AF540910
SAT2	ZIM/34/90	II	BTY3 RS4	Zimbabwe	GU194490
SAT2	ZIM/8/94	II	BTY1 RS3	Zimbabwe	GU194492
SAT2	KEN/8/99	IV	BTY2 RS4	Kenya	AY254730
SAT2	GHA/8/91	V	BTY1 RS3	Ghana	DQ009732
SAT2	SEN/5/75	V	BTY1 RS1 BHK5	Liberia	DQ009738
SAT2	SEN/7/83	VI	CK1 RS1	Senegal	DQ009733
SAT2	SAU/6/00	VII	BTY1 RS1	Saudi Arabia	AY297948
SAT2	ERI/12/89*	VII	BTY2 PK1 RS5	Eritrea	GU194494
SAT2	RWA/2/01*	VIII	PK1 RS1	Rwanda	DQ009730
SAT2	ANG/4/74	XI	BTY3 RS3	Angola	DQ009736
SAT2	UGA/2/02	XII	PK1 RS1	Uganda	DQ009731
SAT2	ZAI/1/74	XII	BTY2 RS4	Zaire	DQ009737

Protective strains are starred.

### Improving estimates for r_1_-values

A key feature of our analysis was to develop a formal approach for including data from multiple sera, experiments and batches. The use of homologous and heterologous titres from the same serum to calculate an r_1_-value controls for between-serum variability [25], and in order to control for between-experiment variation, at least three repetitions are officially advised [6] – when more than one of either the homologous or heterologous VNT is carried out, then the results are usually averaged [30]. Our aim was to go beyond this, and combine all available titres measured in all batches for every pair of protective strain and challenge virus to produce a coherent set of best estimates for all of the true underlying r_1_-values simultaneously.

This was achieved by first determining the presence or absence of and then estimating the magnitude of any consistent inter-experiment, inter-batch or other variability in the data. We built a linear mixed-effects model with log titre of the challenge virus versus protective strain as the response variable. The challenge virus (p = 10^−44^), protective strain (p = 10^−12^) and their interaction (p = 10^−27^) were significant fixed effects, but neither serotype nor whether sera were prepared by vaccination or infection was found to be significant. We would not have expected to see this latter effect since it was confounded with protective strain as all sera for each strain were generated by only one of vaccination or infection. A random effect at the level of experiment accounted for the inter-experiment variability. By comparing models with random effects to allow for other sources of variability, we determined that there was consistent variability between sera (p = 10^−15^), but not between batches (see Materials and Methods). Apart from the one identified above, other interactions between these effects (protective strain, challenge virus, serum and experiment) were not found to be significant. This “best consensus estimate” model could thus be written as:
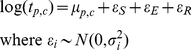
(1)where *t_p,c_* is the titre for a neutralisation test for protective strain p and challenge virus c, *μ_p,c_* is the mean log titre, *ε_S_* and *σ_S_* ^2^ are the best linear unbiased predictor and associated variance for the random effect of serum, *ε_E_* and *σ_E_* ^2^ are the equivalent measures for experiment, and *ε_R_* and *σ_R_* ^2^ are the model residuals and associated variance.

Estimates of these variances were used to examine the expected uncertainties associated with standard methods of estimating r_1_-values. The between-serum variance was 0.072, which gives a 95% confidence interval around the estimate of +/−0.53 log titres due to inter-serum variability; as was noted earlier, this is eliminated by always using homologous and heterologous titres from the same serum to calculate an r_1_-value. However, the remaining (between-experiment and residual) variances sum to 0.287, which gives a 95% confidence interval around the estimate of an *individual serological r_1_-value* (*i.e.* using 1 homologous titre and 1 heterologous titre from the same serum in the same batch, as is usually the case) of +/−1.49 log titres. With test variability this high it is clear that an improved method of estimating r_1_-values that makes use of all available data would be valuable.

**Figure 1:**
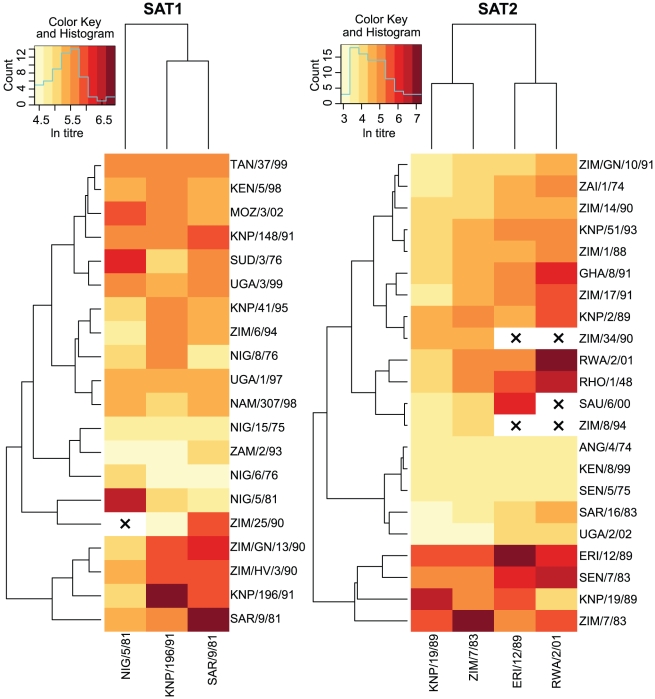
Heatmap and clustering analysis of virus neutralisation titres. Two-dimensional hierarchical clustering of viruses and antisera for SAT1 (left) and SAT2 (right). Viruses were clustered according to their neutralization profiles along the vertical axis. Simultaneously, the antisera were arranged according to their abilities to neutralise the panel of viruses along the horizontal axis. Dendrogram patterns are shown to the left (for viruses) and top (for antisera). The color key for neutralization data points is shown in the histogram in the top left of each plot along with the count of viruses with each titre.×indicates the absence of data.

Estimates of *μ_p,c_* had 95% confidence intervals ranging between +/−0.50 to +/−1.17 log titres, depending on the number of titres available (the greatest uncertainty being associated with r_1_-values estimated from only a single homologous and heterologous titre). These narrower confidence intervals show that our new techniques for estimating *μ_p,c_* provide a substantial improvement on existing methods for the same number of titres. These best consensus estimates of the true means were therefore used as our gold standard for subsequent analyses (Figure 1 and Dataset S1).

### Relating antigenic differences to sequence variation

Structural data were used to identify candidate areas of the capsid that might be antigenically significant (29 and 28 areas for SAT1 and SAT2 respectively – see Materials and Methods for details). These provide the starting point for a related linear mixed-effects approach used to predict r_1_-values from the sequence data and, ultimately, to identify antigenically significant areas of the capsid. The mean log titre, *μ* – the fixed effect term in the estimating model (Equation 1) – was replaced with a predictive term based on differences between capsid-coding sequences of the protective and challenge strains. Removing this fixed effect also necessitated the inclusion of the additional random effect of challenge virus (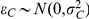, p<10^−10^), and the final model took the form:
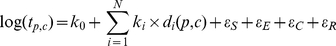
(2)where *k*_0_ is the average titre and *d*_i_ is a raw count of the number of amino acid changes between the protective strain and challenge virus in a single candidate area identified from the structural modelling (the i^th^ out of a total of N areas identified as potentially antigenically significant – see Materials and Methods for the model selection process), with *k*_i_ the regression coefficients.

### Predictive model requirements: Vaccine selection and vaccine coverage

Two different predictions are of interest: first, in an outbreak, the vaccine that best matches a given challenge virus, and second, to judge breadth of coverage of a candidate vaccine strain – the range of r_1_-values that it will produce for selected challenge viruses. Correspondingly, models were validated using two measures of quality of a predictor of antigenic distance: (i) the number of times the protective strain with the predicted highest r_1_-value for a given challenge virus matches the strain that would be selected using the serology data; and (ii) the difference between the predicted r_1_-values for specific pairs of protective strains and challenge viruses and our best consensus estimate r_1_-values for those pairs. In the former case only those challenge viruses that might have appropriate protective strains are considered (we chose those with an estimated r_1_≥0.2) since we are not interested in whether the model correctly chooses the least worst vaccine when none could possibly be effective.

Specifically, candidate models selected by the model-building process were cross-validated by estimating parameters in two different ways, corresponding to our two different requirements: (i) using datasets missing all data for each challenge virus in turn, and comparing the vaccine choice with that obtained using our gold standard estimates; or (ii) using datasets missing all data for each protective strain in turn, and comparing the r_1_-values generated for that missing protective strain compared with our gold standard estimates. The best models for each serotype are reported below.

### Vaccine match prediction for new virus isolates

**Figure 2:**
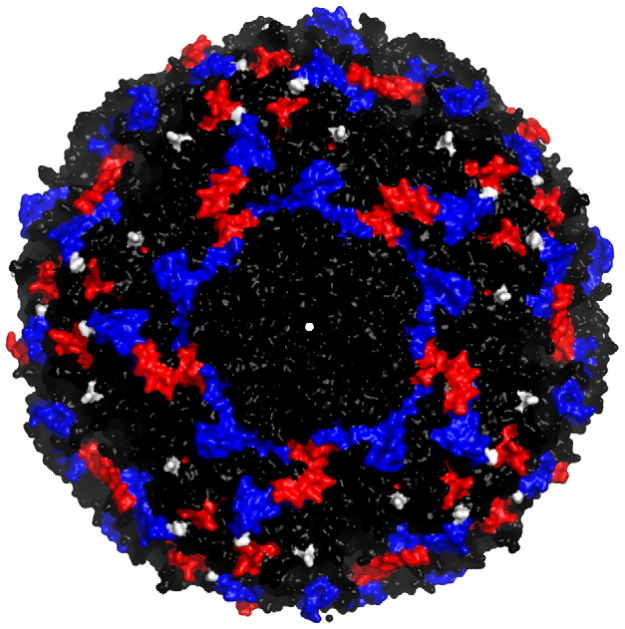
Antigenically significant areas of the SAT1 and SAT2 capsids. The image shows the area around one of the five-fold axes of symmetry of the capsid (centre). Changes to blue areas were used as predictors of loss of cross-reactivity for SAT1, while changes to red areas were used for SAT2. The two residues identified as parts of an epitope – residue 138 on the VP3 E-F loop and residue 198 on the VP2 H-I loop – are coloured white. Note that these two residues and the other areas are repeated multiple times in the image due to structural symmetries in the capsid.

Eighteen out of the 20 SAT1 viruses but only 9 out of the 22 SAT2 viruses had protective strains close enough to offer some cross-reactivity (r_1_≥0.2), and so were included in the cross-validation. For SAT1 the best model after cross-validation contained two terms, the number of amino acid changes in the VP1 G-H loop and beyond (residues 132–174, which contains the major antigenic site for FMDV as well as sites in the H-I loop), and in the VP3 H-I loop (residues 191–202, which contains amino acids identified by MAb escape mutant studies as part of the epitope labelled Site 3 on A10 [24]) – see Figure 2, blue, for visualization. The formula for the r_1_-value predictor is:
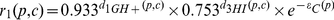
(3)where the *ε_C_* are the best linear unbiased predictors from Equation 2. For SAT2 the best model contained three terms: the number of amino acid changes in the VP1 C terminus (residues 200–224, which contains Site 1b on O, Sites C and D on serotype C, Site 2 for A10, Sites 3 and 4 for A12 and Site 2 for A5 – [21], and references therein), in the VP2 B-C loop (residues 70–82, which contains Site 2 on O, Site 3 on A10, Site 1 on A5 and another part of Site D on C – [21], and references therein), and residue 178 in the VP1 H-I loop (the H-I loop as a whole contains Site 1 for A12 and Site 4 for A10 – [21], and references therein)– see Figure 2, red, for visualization. The formula is:
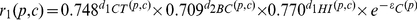
(4)


**Figure 3:**
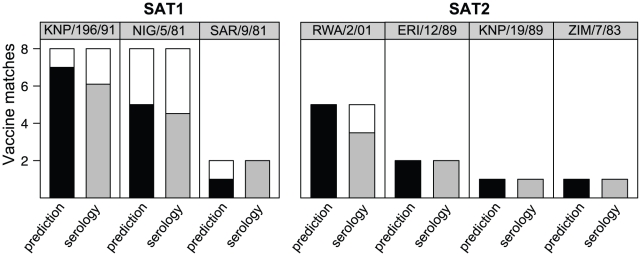
Vaccine matching using sequence data. Charts show the number of times that the best protective strain estimated using the full serological dataset (header) agrees with the predicted best strain using the sequence data (black bars) or the estimated strains using bootstrap samples of individual serological r_1_-values (grey bars). White bars are errors, where the predicted/individual r_1_-values gave a different protective strain.

The predictive models successfully identified 13 SAT1 matches (72%) and all 9 SAT2 matches (100%) (Figure 3). This accuracy is comparable with that obtained using the individual serological measurements, which, by bootstrapping the raw titres to generate individual serological r_1_-values, we estimated would correctly identify the best strain 70% of the time for SAT1 and 83% for SAT2 (a multinomial test on these values shows that there is no significant difference between the serological and predicted values). Though additional serological and sequence data would ultimately improve the predictive model, it currently performs at least as well as standard serological approaches.

### r_1_-value prediction for new vaccine candidates

A small bias was observed for all of the candidate models in their predictions for heterologous titres relative to homologous titres. This does not affect the vaccine match experiments where the aim is to reduce relative error (in the differences between r_1_-values for different protective strains using the same challenge virus) potentially at the expense of absolute error (in the r_1_-values themselves). In this case, however, for accurate r_1_-value prediction the aim is to reduce this absolute error. Adding a term to the models that explicitly distinguished homologous and heterologous titres removed this bias, and so it was included in all of the candidate models.

The best predictive model of r_1_-values following cross-validation for SAT1 contained the same two terms as before (the number of amino acid changes in the VP1 G-H loop and beyond, and the number in the VP3 H-I loop), together with a term that is present when titres are heterologous. The formula is:
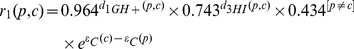
(5)


**Figure 4:**
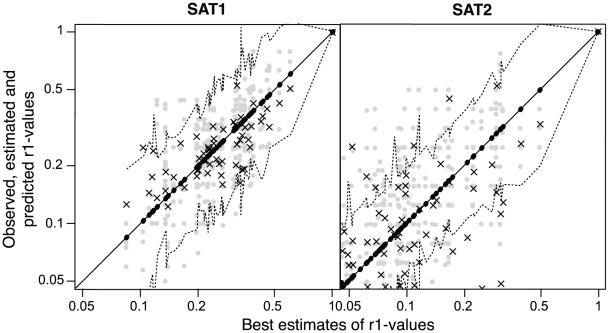
Predicting cross-reactivity using sequence data. Bootstrap samples of individual serological r_1_-values (grey dots), predictions (black crosses) and matching best consensus estimates – our gold standard – and their confidence limits (black dots and dotted lines) against best consensus estimates for SAT1 and SAT2 r_1_-values. Because of the log-normally distributed variance structure of the r_1_-values, data are plotted on a log scale.

Ninety eight percent of the predictions (Figure 4, SAT1, black crosses) are within the 95% confidence limits of the gold standard estimates (dashed lines), which is significantly better than achieved by individual serological r_1_-values (grey dots) at 87% (Fisher's exact test, p<0.01). The variance around the gold standard estimates is significantly lower for the predictions than the individual serological r_1_-values (0.09 rather than 0.18, Bartlett test, p<0.01).

The best predictor of r_1_-values for SAT2 also contained the same three terms as before (the VP1 C terminus, the VP2 B-C loop, and a single residue in the VP1 H-I loop), again together with a term for heterologous titres. The formula is:
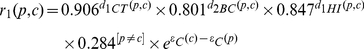
(6)


For SAT2, 77% of the predicted r_1_-values were within the confidence limits of the gold standard estimates (Figure 4, SAT2, black crosses), which is not significantly different from 66% for individual serological r_1_-values (grey dots). Variances were also not significantly different (0.40 compared to than 0.43). Both predictions and serological measurements are less accurate than those for SAT1 due to the lower repeatability of SAT2 serology (Figure 4, grey dots).

### Identifying epitopes by controlling for phylogenetic structure

The above predictive models identify those areas that are correlated with loss of cross-reactivity. To identify those areas that are directly responsible for antigenic variability it is necessary to develop models that additionally control for the phylogenetic relationships between virus strains. The phylogenetic control extends Equation 1 in an analogous manner to the predictive model (Equation 2):
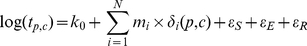
(7)where *δ_i_* is a delta function which is 1 if p and c are separated by branch *i* of the phylogenetic tree and 0 otherwise. Loss of cross-reactivity is caused by amino acid substitutions in the capsid proteins, and any individual substitution must occur in a specific branch of the phylogenetic tree (though we may not be able to determine which). Each branch partitions the tree into two groups, and where a branch effect represents changes that impact significantly on cross-reactivities, they will be higher between viruses within the groups than those between groups (after controlling for other effects). For instance, where a terminal branch is identified, the fixed effect of that branch specifies an amount by which the virus to which it leads (the first group) is antigenically distant from all other viruses (the second group). A significant internal branch, similarly, identifies a clade that is antigenically distant from the rest of the tree. By building a model containing all of the branches in the tree, and then using a stepwise elimination procedure to remove branches which do not significantly improve the model fit (p>0.05), we are left with the set of branches that, when traversed, significantly account for reductions in antigenic cross-reactivity.

**Figure 5:**
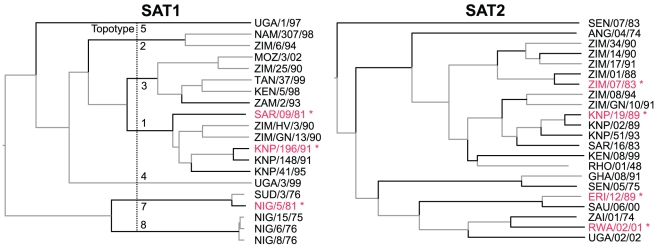
Phylogenetic trees indicating the branches controlled for in the analysis. SAT1 and SAT2 phylogenetic trees, showing protective strains (red, starred) and branches associated with significant drops in antigenic cross-reactivity (black lines, p<0.05). Topotypes are shown for SAT1. The origin and passage history of each virus is described in Table 1.

Twelve phylogenetic branches are significant in SAT1 and twenty-one in SAT2 (black lines, Figure 5). For SAT1 these are six branches that each partition individual topotypes from the rest of the tree, and five terminal branches that lead to viruses for which large numbers of titres have been obtained (including the three protective strains) as well as one that is antigenically very distant from the protective strains (ZAM/2/93, which has no r_1_-value above 0.2). For SAT2, there are six internal branches throughout the tree and fifteen terminal branches leading to ten of the thirteen viruses that are antigenically distinct (again, all r_1_-values are below 0.2) and five other viruses (including the four protective strains).

Any model containing these terms controls as completely for the phylogeny as is possible with the data available, and should a model have a significantly better fit than the phylogenetic model on its own, it must achieve this by some mechanism other than phylogenetic correlation. A simple combination of Equations 2 and 7 provides a potential model, with an additional term for the raw count of the number of amino acid changes between the protective strain and challenge virus in a single candidate area:
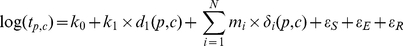
(8)where *d*_1_ is the count of substitutions at a specific site, and *k*_1_ the associated regression coefficient. The phylogenetic control terms account for repeated measurement of all significant shared phylogenetic history. However, in doing so, they remove all significant direct effects of substitutions at individual branches of the tree, but are not designed to capture the interactions involved in multiple and/or convergent substitutions at the same sites in different branches. Consequently, the substitution count in any area significantly improves the model if it corresponds to this substitution structure. Parallel and/or back-mutations, relatively frequent in such highly variable viruses, are therefore strong signals used by the model to determine antigenically significant areas. The phylogenetic control is therefore conservative in that significant sites with substitutions at only one branch in the tree will not be identified, as the different substitutions in that branch cannot be readily disambiguated. Nevertheless, after controlling for phylogeny, the twenty-nine SAT1 areas tested with the model were collectively significant predictors (p<0.05 [31]), but the twenty-eight areas for SAT2 were not significant (collectively or individually).

Because substitutions are ultimately responsible for the loss of cross-reactivity, the substitutions contributing to counts in these SAT1 areas must be responsible for this loss unless they are co-occurring with causative substitutions. Any such causative substitutions should, however, be identifiable because substitution counts in areas containing them will improve the model fit. Comparing the individually best SAT1 areas from above with bootstrapped random sequences of the same length from other parts of the capsid, however, fails to identify other causative substitutions, and eight areas were instead found to be significant after a Holm-Bonferroni correction for the number of terms [32] (p<10^−12^ collectively). Of these eight terms, seven were individually significant in the previous test (p<0.05). These seven terms consisted of five that corresponded exactly to the five areas identified as the constituent parts of the Site 3 conformational epitope for A10 [24] (p<10^−8^ collectively), one was the VP1 G-H loop (p<0.001), and the last was the VP3 G-H loop, previously identified as Site 3 on A12 [18] (p<0.01).

To identify the specific residues responsible for these drops in antigenic cross-reactivity, Equation 8 is trivially modified to test substitutions to the 62 individually variable residues in the seven areas identified (instead of the 29 candidate areas). Again, this is a conservative test, as it will only identify residues where multiple/convergent substitutions occur at different branches in the phylogeny. Collectively, changes to the residues are significant after controlling for phylogeny (p<0.005), but only two residues are individually significant (p<0.05). Bootstrap comparisons with other residues showed these to be the two most significant predictors of loss of cross-reactivity out of all the residues in the capsid, and both are adjacent to residues identified by MAb escape mutant studies on A10 as part of Site 3 [24]. These were residue 138 on the VP3 E-F loop and residue 198 on the VP2 H-I loop – see Dataset S2 for alignment, and Figure 2, white, for visualization – and the expected effects of substitutions at those residues are a reduction in cross-reactivity of 25% (95% CI 8%–40%) and 16% (95% CI 0%–30%) respectively.

To test whether areas and individual residues vary in their significance across the whole serotype or are conserved, a random effect (*κ(r)* in Equation 9) that allows the count (*k*_1_ in Equation 8) to vary in significance for different protective strains, challenge viruses or sera (*r*) can be added to the model:
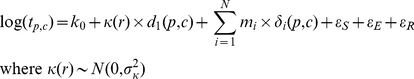
(9)


For SAT1 none of these effects improve the model (p>0.1 for all areas and individual residues). For SAT2, however, although no areas or residues are significant individually, thirty-one of them have significant interactions (p<0.05) with at least one of these ‘*r*’ terms, which suggests that there may be some variability in the significance of parts of the capsid for loss of cross-reactivity within the serotype.

## Discussion

The identification of antigenic sites on individual FMDV isolates is time consuming, with the consequence that data are not available for all serotypes, much less for all isolates. Indeed, very little is known about the important epitopes for SAT1 and SAT2 viruses, impacting on the potential to both design vaccines with broader or better targeted antigenic cover and predict the efficacy of a particular vaccine strain against circulating viruses in the field. We have identified seven areas containing what we believe to be three epitopes for SAT1, and we provide evidence that these are conserved across our whole sample. We have further quantified the effect of substitutions at two specific residues in one of these epitopes. The conservative phylogenetic control employed throughout the analysis means that this may not be an exhaustive list of antigenically significant areas of the capsid, and is almost certainly not for residues, as it will only identify ones where multiple/convergent substitutions occur at different branches in the phylogeny. The areas that are identified do, however, correspond to epitopes identified by MAb escape mutants for other serotypes, and both of the specific residues found are (after alignment) adjacent to ones which are part of Site 3 on A10 [24]. Confirmatory evidence that the phylogenetic control is acting as expected is provided by the fact that for SAT1 all of the internal branches that are identified as antigenically significant correspond to previously identified antigenically important events, that is to say branches that partition individual topotypes from the rest of the tree. For SAT2, we have identified variability in the significance of different sites for different protective and challenge strains, and even different sera, that may indicate epitopes are present on some viruses but not on others. This may help to explain the much greater observed antigenic variability in SAT2 compared to SAT1 despite their similar genomic variability [11], as well as the absence from our analyses of identifiable epitopes that are conserved across the serotype.

We have also used cross-reactivity data generated for SAT1 and SAT2 FMDV to develop a linear mixed-effects model that uses replicates and repeated experiments to more accurately account for variability in measurement and so generate better estimates of cross-reactivity for FMDV. We have enhanced this model with sequence and structural data to identify surface-exposed residues that correlate with loss of cross-reactivity and then built models using counts of amino acid substitutions in selected areas to predict cross-reactivity. We note that all of the areas used in these models are associated with epitopes identified by MAb escape mutants for other serotypes. Furthermore, for SAT1 they also correspond to parts of the new epitopes identified above.

These predictive models were used to successfully identify the efficacy of novel candidate vaccines against the virus isolates, with 98% of SAT1 predictions within the 95% confidence intervals for our gold standard estimator of true r_1_-values, and 77% of SAT2 predictions. For SAT1 this was significantly better than individual serological r_1_-values despite the predictions being made without the use of any sera from the protective strains. Related models were also used to predict the best vaccine match for novel virus isolates. For the 9 SAT2 virus isolates for which any match existed in the data (r_1_≥0.2), the model correctly predicted all 9, and for the 18 SAT1 isolates, the model predicted 13. In both cases there is no significant difference between the model predictions and the serological results. The uncertainty inherent in the VNT and the variability between different experiments balance any inaccuracies in the predictive model, making it at least as effective a measure of r_1_-value and vaccine match as serology itself unless the latter is repeated multiple times. Improving the serological tests is an area of active research [33], and we anticipate being able to improve the predictive models further by exploiting such improved serological datasets.

The accuracy of the r_1_-value predictions and the inaccuracy of the matching for SAT1 (relative to SAT2) may have the same cause – two of the anti-sera were raised against viruses of the same topotype (topotype 1 – constituting 70% of the titres), producing a good model for that topotype, but with little power to generalise and predict cross-reactivity for significantly different viruses clustering in other topotypes. Consequently, it identified 8 out of 10 correctly when the answer was a topotype 1 vaccine, but only 5 out of 8 for the other topotype. Conversely, the relative inaccuracy of the r_1_-values but the accuracy of the matching for SAT2 may share the opposite cause – 4 anti-sera were raised against 4 different topotypes, giving a better estimate of which areas were antigenically significant in general and thereby allowing better vaccine matching. However, because of the relative sparsity of data from any individual topotype (there were at most 92 titres for any individual topotype for SAT2, against 170 for SAT1), we obtained a poorer estimate of the relative importance of each area, and therefore a less accurate r_1_-value prediction. The greater inherent variability in SAT2 titres also necessitated more data to acquire an accurate estimate. These complementary results suggest that to refine the model further more data from different topotypes will improve the vaccine matching in SAT1, and more from the same topotypes will improve the estimates of cross-reactivity in SAT2.

The VP1 G-H loop is known to contain major epitopes in all serotypes of FMDV where MAb studies have been conducted; substitutions in it are therefore considered to be a significant determinant of loss of cross-reactivity. Our SAT1 epitope analysis identified this loop and the model used it to predict cross-reactivity. However, our SAT2 model did not find the number of amino acid substitutions in the candidate area containing the loop to be in general a good correlate. There are three potential explanations. First, the G-H loop has a high substitution rate, so high indeed that we suggest that the epitope(s) may very often not cross-react even between closely related strains, giving the model little data with cross-reacting epitopes from which to identify a pattern. Second, the candidate area is much bigger than just the G-H loop, therefore including many residues that are not antigenically significant. Finally saturation can occur, causing the actual count even inside the G-H loop to cease to be meaningful. The combination of these effects makes it very difficult to detect the signal of epitope loss from the noise of other substitutions.

Better predictors might be obtained by selecting smaller candidate areas. However, there is a risk of “fishing” until an appropriate sub-sequence is discovered, leaving the generality of the technique uncertain. Related work on influenza A [34], [35], which was the first to attempt this kind of prediction, examined 101 residues (chosen based on previous laboratory identification of antigenic sites) and built models containing up to 19 terms, testing orders of magnitude more models than our 2-/3-term out of 28-/29-area models. The advantage of the approach taken here is that the areas to be examined were determined by a single *a priori* criterion, the strategy for choosing models to test was fixed in advance, and the relatively small number of models examined in the model-building process was easily controlled for by a simple statistical correction.

A further strength of our approach is that it identifies antigenically significant areas. These can be validated entirely independently through comparison with MAb escape mutant-derived epitope information. Of the 5 areas identified here, all have been identified by previous MAb work in other serotypes. Furthermore, the two SAT1 areas are specifically identified in separate analyses as containing epitopes for this serotype, which provides further strong evidence that the model is indeed predictive rather than merely correlative. For SAT2 the evidence is weaker since the epitopes have only been identified on other serotypes, and the evidence that there may be epitopic variability within the serotype suggests that more caution should be applied in using them to predict the cross-reactivity of distantly related isolates.

This work could be further validated by a reverse-genetics approach targeting the specific SAT1 residues identified as antigenically significant. Our methodology could be used to evaluate the effect of amino acid substitutions by predicting coverage against a panel of circulating viruses, allowing potential vaccine candidates that are expected to better match the panel to be easily identified. Interestingly, because of the high substitution rate obscuring any signal in the VP1 G-H loop, identified substitutions would probably not be located in the major antigenic site of FMDV but instead be found in other antigenically important areas.

The technique developed here can be used directly for any FMDV serotype and potentially for any similar virus where cross-reactivity, sequencing and structural studies have been carried out, both to identify epitopes, and to predict vaccine match for new isolates and estimate efficacy of new candidate seed strains. This can be done by exploiting historical datasets, and is therefore a quick, low cost and valuable method for better understanding antigenic relationships. In summary, the use of sequence data to predict antigenic relationships is a powerful tool that has the potential to be applied to a variety of different infectious agents.

## Materials and Methods

### Ethics statement

All procedures were approved by the Onderstepoort Veterinary Institute Animal Ethics Committee according to national animal welfare standards.

### Virus isolates, RT-PCR and nucleotide sequencing

The viruses were either supplied by the World Reference Laboratory for FMD at the Institute for Animal Health, Pirbright (United Kingdom) or form part of the virus databank at the Transboundary Animal Diseases Programme (TADP), Onderstepoort Veterinary Institute (South Africa). Viral RNA was extracted from cell-culture-adapted isolates and cDNA synthesised [36]. The sequences for the P1-2A-coding regions were obtained via RT-PCR of viral genomic RNA using existing primers [36]–[39]. Direct DNA sequencing of the P1-2A region derived from a given FMDV isolate yielded a master sequence representing the most probable nucleotide for each position of the sequence. Due to the quasispecies nature of FMDV populations, polymorphisms were detected in some nucleotide positions. Nevertheless, all positions could be unambiguously assigned to a single nucleotide due to the high degree of redundancy generated by a genome-walking approach. Contigs for the *ca*. 2.2kb region were compiled using Sequencher™ vs4.7 (Gene Codes Corporation). Since these are protein-coding regions, the amino-acid sequences were aligned with ClustalW (v.1.83) and this alignment was then used to align the nucleic-acid sequences.

### Animal sera and virus neutralisation test

The antigenic diversity of the field isolates was determined using virus neutralisation assays in micro-titre plates using IB-RS-2 cells as the indicator system [25]. Cattle sera against reference SAT1 and SAT2 viruses were prepared by two consecutive vaccinations (vaccinated at day 0, boosted at day 28 and bled at day 38) using the following vaccine strains: SAT1: SAR/9/81 and KNP/196/91 (both topotype 1, see Table 1); SAT2: ZIM/7/83 (topotype II) and KNP/19/89 (topotype I) or convalescent sera obtained from 21 days post-infected cattle for SAT1: NIG/5/81 (topotype 7); SAT2: RWA/2/01 (topotype VIII) and ERI/12/89 (topotype VII). Cattle were housed in the isolation facility at TADP and all procedures were approved by the Onderstepoort Veterinary Institute Animal Ethics Committee. The neutralisation assays were performed against viruses from the various topotypes as indicated in Table 1 after their adaptation on IBRS-2 cells. The end point titre of the serum against homologous and heterologous viruses was calculated as the reciprocal of the last dilution of serum to neutralise 100 TCID_50_ in 50% of the wells(*ibid.*).

### Structure

The crystal structure of the SAT1 BOT/1/68 capsid was solved at a resolution of 3Å (Fry et al., unpublished) (Protein Data Bank ID: 2wzr, r2wrsf). This is the only structure in existence for any SAT1 or SAT2 virus. SAT1 amino-acid sequence alignments were compiled with ClustalW. Structures were visualised and the surface-exposed residues identified with the PyMol Molecular Graphics System v1.2r0 (DeLano Scientific LLC). Exposed regions of SAT2 were approximated by alignment with the SAT1 structure.

The aligned SAT sequences were classified according to whether they coded for surface-exposed residues or not as determined from the above structure. Those that did were grouped into the longest possible contiguous sub-sequences where all of the residues were surface exposed. Forty-three such sub-sequences were found, which broadly corresponded to the loops and termini of the VP1, VP2 and VP3 proteins, though some loops were not exposed, and some divided into more than one sub-sequence separated by hidden sections. This division was chosen as the simplest way of breaking the full sequence down into a number of *candidate areas* each of which might be implicated in one or more antigenic site(s). Of these, 14 of the sub-sequences were invariant in SAT1, as were 15 of the areas in SAT2, leaving 29 and 28 areas respectively in the two serotypes to test as possible predictors (see Dataset S2 for the areas identified).

Although the areas identified for SAT2 were only an approximation to the true surface exposed areas for that serotype, residues from every known epitope of FMDV were contained within these regions [14]–[24], and so the areas are likely to be sufficient.

### Phylogenetic analysis

A phylogenetic tree was generated from the nucleotide sequence data using a relaxed uncorrelated exponential clock, and a GTR+CP_112_+Γ_112_+I nucleotide model [40]. This was identified as the best model using Bayes Factor analysis [41], although all similar models produced the same tree topology. All of these models and analyses are included in BEAST version 1.5.3 and Tracer version 1.5.0 [42].

### Data

The experimental variables used in this study are described below.

- **Protective strain**: the virus strain against which each animal has been previously vaccinated or with which it has been infected;
- **Vaccination status**: whether the animal acquired protection through vaccination (1) or infection (0);
- **Serum**: each serum is drawn from a single animal, so serum labels correspond to individual donor animals;
- **Challenge virus**: virus isolate used in neutralisation experiment;
- **Serotype**: serotype of the protective (and challenge) strain;

The neutralisation tests were grouped into *experiments* where the same serum was used at the same time with the same challenge virus (tests within an experiment are *replicates*); different experiments were grouped into *batches* by the time at which they were performed. Possible variability at these levels was investigated when building the model. Our gold standard best consensus estimates of r_1_-values are available in Dataset S1; raw serological data is available on request.

### Statistical modelling and model selection

There were five stages to the statistical modelling:

1. First, a linear mixed-effects model [43] was built with log titre of the VNTs as the response variable (which are normally distributed – Lilliefors normality test, p>0.5) – using raw titres gave us neither normally-distributed (p<10^−15^) nor homoscedastic residuals [30] – using the R statistical software [44] and the modelling package lme4. This model allowed accurate estimation of r_1_-values from serological data.
2. The fixed effects in this model (Equation 1) were then replaced with sequence-based predictors (a selection of counts in candidate areas and the count of total amino acid substitutions were used to test the model), and the model selection approach outlined below used to generate a model that predicted cross-reactivity directly from sequence data (Equations 2–6).
3. These were replaced in turn with phylogeny-based effects (see below) to control for the phylogenetic structure of the data (Equation 7).
4. Individual areas and residues were added to this model to identify epitopes (Equation 8).
5. Finally, within-serotype variability in epitopes was investigated (Equation 9).

#### Model selection

The predictive models (Equation 2) were generated by sequentially adding the count of non-synonymous changes in each of the candidate antigenic areas as a fixed effect in a standard stepwise regression which continued for a variable number of steps until no further terms could be added. Specifically, the probability that each model was a significantly better predictor than its precursor was assessed by a likelihood ratio test since the models were nested. For multiple tests each with p-values p_i_, the statistic −2 Σ log p_i_ is expected to be χ^2^ distributed with twice as many degrees of freedom as tests under the null hypotheses [31]. When this was not the case (p<0.05), then the best predictors that were individually significant (after a Holm-Bonferroni correction for the number of terms [32]) were used as bases for the next step of the regression. The stepwise regression technique was repeated until no more terms could be added to form a small set of candidate models. The best of the final models were then cross-validated. Details of the best models are found in the results (Equations 3–6).

#### Controlling for phylogeny

Amino acid substitutions on the capsid (including those identified above) are correlated with antigenic distance; this could be a direct relationship or may arise indirectly via relationships between substitutions, phylogenetic history and antigenic drift, as is found in influenza A [45]. Neglecting to control for evolutionary history has caused false positive rates of between 20 and 40% in similar analyses [46]; these arise because substitutions that constitute the shared history of virus pairs have only occurred once and therefore constitute only a single independent piece of evidence that these substitutions are important. To account for these repeated measures it is necessary for us to implement phylogenetic control.

However, existing mechanisms for controlling for phylogeny focus on properties (or traits) of the leaves of tree and not the relationships between them [47]. Indeed it is these relationships (the *contrasts*) that are used to control for the evolutionary history, whereas for us these are the signal – the cross-reactivity. We wish instead to identify the causes of the changes in cross-reactivity while controlling for the common evolutionary history.

In practice, each branch on the phylogenetic tree (see above) represents a set of common substitutions by which any pair of viruses either side of the branch differ (unless multiple and/or convergent substitutions have occurred). Any comparison of antigenicity between two viruses either side of the branch will be affected by those changes. A fixed effect is therefore added to the model for each branch (*δ*_i_, Equation 7); this is non-zero if the branch is travelled (and thus these changes have occurred) in the traversal of the tree between the protective strain from which the serum is derived and the virus isolate in a cross-reactivity test. Including these terms in the analysis controls for repeated measures of this traversal.

Because of the necessarily limited number of protective strains we do not explore every path through the tree, and so there is some ambiguity in the allocation of weights to branches (essentially we have more unknowns than equations). These ambiguities mean that the models cannot be used predictively, but this does not prevent their use for phylogenetic control.

Model development begins by constructing a model with the maximal set of fixed effects; these are then removed using stepwise regression until all of those left significantly improve the model fit (p<0.05). In this manner we have controlled for repeated measures of every significant piece of shared phylogenetic history. Because the phylogenetic trees are different for the two serotypes the serotypes are modelled separately.

#### Identifying epitopes

The phylogenetic control terms account for repeated measurement of all significant shared phylogenetic history. However, in doing so they remove all significant direct effects of substitutions at individual branches of the tree. Consequently, the substitution count in any area can only significantly improve the model if it corresponds to multiple and/or convergent substitutions at the same sites in different branches. Modelling phylogenetic control in this way therefore provides a conservative estimate of the number of areas that directly affect antigenicity.

Sequence-based predictors were added to the models – again, substitution counts for each of the candidate areas identified in the structural analysis – to determine which sub-sequences were the best predictors after controlling for phylogeny (*d*_1_, Equation 8), and these were then compared to bootstrapped samples from the remaining capsid surface (randomly assembled sub-sequences of the same length as each candidate). Since changes to the capsid proteins must be responsible for loss of cross-reactivity, comparing specified sub-sequences to the rest of the capsid after controlling for phylogenetic structure directly determines whether these areas contain true predictors or whether they contain correlates, with the true epitopes being found elsewhere. To identify the individual constituent residues of epitopes, exactly the same mechanism is used on individual residues instead of areas.

#### Detecting within-serotype variability

In order to determine whether there was variability in the effects of substitutions at specific sites within a serotype, we added a random effect that allows the effect of the count to vary for different protective strains, challenge viruses or sera (Equation 9). Should one of these terms be found to improve model fit, this would provide evidence that there is variability across the phylogeny as to the importance of different sites.

## Supporting Information

Dataset S1 Best consensus estimates of all r_1_-values used in the study.
(0.03 MB XLS)

Dataset S2 Reference alignment of the protective strains to the study isolates. The dataset shows the VP2, VP3 and VP1 proteins of the protective strains after alignment to all of the SAT1 and SAT2 isolates used in the study. The 43 contiguous surface-exposed areas identified by the capsid structural analysis are highlighted. Areas with no amino-acid variability are in grey, and areas with variability are in red for the serotypes for which there is variation.
(0.14 MB DOC)
