# Supplementary material for: Sequence-Based Prediction for Vaccine Strain Selection and Identification of Antigenic Variability in Foot-and-Mouth Disease Virus
Source: PLoS Comput Biol. 2010 Dec 9;6(12):e1001027. doi: 10.1371/journal.pcbi.1001027 (PMC3000348; doi:10.1371/journal.pcbi.1001027)
Supplement: Dataset S2 — Reference alignment of the protective strains to the study isolates. The dataset shows the VP2, VP3 and VP1 proteins of the protective strains after alignment to all of the SAT1 and SAT2 isolates used in the study. The 43 contiguous surface-exposed areas identified by the capsid structural analysis are highlighted. Areas with no amino-acid variability are in grey, and areas with variability are in red for the serotypes for which there is variation. (0.14 MB DOC) [file pcbi.1001027.s002.doc]

| VP2 | | 1 2 3 4 5 |
| --- | --- | --- |
|  |  | 12345678901234567890123456789012345678901234567890 |
| 0 | SAT1 KNP/196/91 | DKKTEETTLLEDRILTTSHGTTTSTTQSSVGITYGYADSDRFLPGPNTNG |
|  | SAT1 SAR/9/81 | DKKTEETTLLEDRILTTSHGTTTSTTQSSVGVTYGYAESDHFLPGPNTNG |
|  | SAT1 NIG/5/81 | DKKTEETTLLEDRILTTSHGTTTSTTQSSVGVTYGYASSDKFLPGPNTNG |
|  | SAT2 KNP/19/89 | DKKTEETTLLEDRIVTTRHGTTTSTTQSSVGVTYGYADADSFRPGPNTSG |
|  | SAT2 ZIM/7/83 | DKKTEETTLLEDRIVTTRHGTTTSTTQSSVGITYGYADADSFRPGPNTSG |
|  | SAT2 ERI/12/89 | DRKTXETTLLEDRILTTRHGTTTSTTQSSVGATLGYADADSFRPGPNTSG |
|  | SAT2 RWA/2/01 | DKKTEETTLLEDRILTTRHGTTTSTTQSSVGITYGYADSDSFKPGPNTSG |
| 50 | SAT1 KNP/196/91 | LETRVEQAERFFKHKLFDWTLEQRFGTTHVLELPTDHKGIYGQLVDSHSY |
|  | SAT1 SAR/9/81 | LETRVEQAERFFKHKLFDWTLEQQFGTTHILELPTDHKGIYGQLVDSHSY |
|  | SAT1 NIG/5/81 | LETRVEQAERYFKQKLFDWDTTQQFGVTHVLELPTDHKGVYGQLVDSYTY |
|  | SAT2 KNP/19/89 | LETRVQQAERFFKEKLFDWTPEKPFGTLYVLELPKDHKGIYGSLTEAYTY |
|  | SAT2 ZIM/7/83 | LETRVEQAERFFKEKLFDWTSDKPFGTLYVLELPKDHKGIYGSLTDAYTY |
|  | SAT2 ERI/12/89 | LETRVQQAERFFKEKLFDWTSDKPFGTLYVLELPKDHKGIYGKLTDSYAY |
|  | SAT2 RWA/2/01 | LETRVEQAERFFKEKLFDWTSDKPFGTLYVLELPKDHKGIYGKLTDSYTY |
| 100 | SAT1 KNP/196/91 | IRNGWDVEVSATATQFNGGCLLVAMVPELCKLSEREKYQLTLFPHQFLDP |
|  | SAT1 SAR/9/81 | IRNGWDVEVSATATQFNGGCLLVAMVPELCKLADREKYQLTLFPHQFLNP |
|  | SAT1 NIG/5/81 | MRNGWDVQVSATATQFNGGCLLVAMVPELCKLDAREKYQLTLFPHQYINP |
|  | SAT2 KNP/19/89 | MRNGWDVQVTATSTQFNGGSLLVAMVPELCSLRDREEFQLSLYPHQFINP |
|  | SAT2 ZIM/7/83 | MRNGWDVQVSATSTQFNGGSLLVAMVPELCSLKDREEFQLSLYPHQFINP |
|  | SAT2 ERI/12/89 | MRNGWDVQVSATSTQFNGGSLLVAMVPELCSLKSREEFQLTLYPHQFINP |
|  | SAT2 RWA/2/01 | LRNGWDVQVSATSTQFNGGSLLVVMVPELCSLKKREEFQLTLYPHQFINP |
| 150 | SAT1 KNP/196/91 | RTNTT--AHIQVPYLGVDRHDQGTRHKAWTLVVMVVAPYTNDQTIGSNKA |
|  | SAT1 SAR/9/81 | RTNTT--AHIQVPYLGVDRHDQGTRHKAWTLVVMVVAPYTNDQTIGSTKA |
|  | SAT1 NIG/5/81 | RTNTT--AHIQVPYLGVDRHDQGKKHKAWTLVVMVVAPYTNDQTIGSSKA |
|  | SAT2 KNP/19/89 | RTNTT--AHIQVPYLGVNRHDQGKRHQAWSLVVMVLTPLTTETQMNSGTV |
|  | SAT2 ZIM/7/83 | RTNTT--AHIQVPYLGVNRHDQGKRHQAWSLVVMVLTPLTTEAQMQSGTV |
|  | SAT2 ERI/12/89 | RTNTT--AHIQVPYLGVNRHDQGKRHQAWSLVVMVLTPLTTEAQMNSGTV |
|  | SAT2 RWA/2/01 | RTNTT--AHIQVPYLGVNRHDQGKRHQSWSLVVMVLTPLTTEAQMNSGTV |
| 200 | SAT1 KNP/196/91 | EVYVNIAPTNVYVAGEKPAKQ |
|  | SAT1 SAR/9/81 | EVYVNIAPTNVYVAGEKPAKQ |
|  | SAT1 NIG/5/81 | EVYVNIAPTNVYVAGERPAKE |
|  | SAT2 KNP/19/89 | EVYANIAPTNVFVAGEKPAKQ |
|  | SAT2 ZIM/7/83 | EVYANIAPTNVFVAGEKPAKQ |
|  | SAT2 ERI/12/89 | EVYANIAPTNVVVAGELPGKQ |
|  | SAT2 RWA/2/01 | EVYANIAPTNVYVAGELPGKQ |

| VP3 | | 1 2 3 4 5 |
| --- | --- | --- |
|  |  | 12345678901234567890123456789012345678901234567890 |
| 0 | SAT1 KNP/196/91 | GILPVAVSVGYGGFQNTDPKTSDPVYGHVYNPARTGLPGRFTNLLDVAEA |
|  | SAT1 SAR/9/81 | GILPVAVSDGYGGFQNTDPKTSDPVYGHVYNPARTGLPGRFTNLLDVAEA |
|  | SAT1 NIG/5/81 | GIVPVAVADGYGGFQNTDPKTSDPIYGHVYNAARTGYPGRYTNLMDVAEA |
|  | SAT2 KNP/19/89 | GIIPVACSAGYGGFQNTDPKTADPIYGYVYNPSRNDCHGRYSSLLDVAEA |
|  | SAT2 ZIM/7/83 | GIIPVACFDGYGGFQNTDPKTADPIYGYVYNPSRNDCHGRYSNLLDVAEA |
|  | SAT2 ERI/12/89 | GIVPVAAADGYGGFQNTDPKTADPIYGYVYNPSRNDCHGRFSNLMDVAEA |
|  | SAT2 RWA/2/01 | GIVPVACADGYGGFQNTDPKSADPIYGHVYNPPRSDCHGRFSNLLDVAEA |
| 50 | SAT1 KNP/196/91 | CPTLLDFN-GVPYVTTQANSGSKVLTCFDLAFGHKNLKNTFMSGLAQYYT |
|  | SAT1 SAR/9/81 | CPTFLDFN-GVPYVTTQSNSGSKVLTRFDLAFGHKNLKNTFMSGLAQYYA |
|  | SAT1 NIG/5/81 | CPTFLDFN-GVPYVTTQSNSGSKVMALFDLAFGHKNLKNTFLSGLAQYYT |
|  | SAT2 KNP/19/89 | CPTFLNFD-GKPYVVTKNN-GDKVMTCFDVAFTHKVHKNTFLAGLADYYT |
|  | SAT2 ZIM/7/83 | CPTFLNFD-GKPYVVTKNN-GDKVMTCFDVAFTHKVHKNTFLAGLADYYA |
|  | SAT2 ERI/12/89 | CPTLLNFD-GKPYVVTKNN-GDKVMASFDVAFTHKVHKNTFLAGLADYYT |
|  | SAT2 RWA/2/01 | CPTLLNFD-GKPYVVTKNN-GDKVMAAFDVAFTHKVHKNTYLAGLADYYT |
| 100 | SAT1 KNP/196/91 | QYSGTLNLHFMYTGPTNNKAKYMVAYIPPG--THPLPETPEMESHCHHAE |
|  | SAT1 SAR/9/81 | QYSGTLNLHFMYTGPTNNKAKYMVAYIPPG--THPLPETPEMASHCYHAE |
|  | SAT1 NIG/5/81 | QYSGTLNLHFMYSGPTNNKAKYMVAYIPPG--THPLPRTPEQASHCYHAE |
|  | SAT2 KNP/19/89 | QYQGSLNYHFMYTGPTHHKAKFMVAYIPPGIETEKLPKTPEDAAHCYHSE |
|  | SAT2 ZIM/7/83 | QYQGSLNYHFMYTGPTHHKAKFMVAYIPPGIETDRLPKTPEDAAHCYHSE |
|  | SAT2 ERI/12/89 | QYSGSLNYHFMYTGPTHHKAKFMVAYVPPGTELHTLPQTPEDAAHCYHAE |
|  | SAT2 RWA/2/01 | QYSGSLNYHFMYTGPTHHKAKFMVAYVPPGIQTSDLPRTPEDAAHCYHSE |
| 150 | SAT1 KNP/196/91 | WDTGLNSTFTFTVPYVSAADFAYTYSDEPEQASVQGWVGVYQVTDTHEKD |
|  | SAT1 SAR/9/81 | WDTGLNSTFTFTVPYVSAADYAYTYSDEPEQASVQGWVGVYQVTDTHEKD |
|  | SAT1 NIG/5/81 | WDTGLNSTFTFTVPYVSGADFAYTHTDEPEQASVQGWVGVYQITDTHEKD |
|  | SAT2 KNP/19/89 | WDTGLNSQFTFAVPYVSASDFSYTHTDTPAMATTNGWVAVYQVTDTHSAE |
|  | SAT2 ZIM/7/83 | WDTGLNSQFTFAVPYVSASDFSYTHTDTPAMATTNGWVAVFQVTDTHSAE |
|  | SAT2 ERI/12/89 | WDTGLNSSFSFAVPYLSAADFSYTHTDTPAMATTNGWVVVLQVTDTHSAE |
|  | SAT2 RWA/2/01 | WDTGLNSNFTFAVPYLSSADYSYTHTDTPAMATTNGWVVVLQVTDTHSAE |
| 200 | SAT1 KNP/196/91 | GAVVVSVSAGPDFEFRMPISPSRQ |
|  | SAT1 SAR/9/81 | GAVVVSISAGPDFEFRMPISPSRQ |
|  | SAT1 NIG/5/81 | GALIVTVSAGPDLEFRLPISPSRQ |
|  | SAT2 KNP/19/89 | AAVVVSVSAGPDLEFRFPIDPVRQ |
|  | SAT2 ZIM/7/83 | AAVVVSVSAGPDLEFRFPVDPVRQ |
|  | SAT2 ERI/12/89 | AAVVVSVSAGPDLEFRFPVDPVRQ |
|  | SAT2 RWA/2/01 | AAVVVSVSAGPDLEFRFPIDPVRQ |

| VP1 | | 1 2 3 4 5 |
| --- | --- | --- |
|  |  | 12345678901234567890123456789012345678901234567890 |
| 0 | SAT1 KNP/196/91 | TTSAGEGAEPVTTDASQHGGDRRTT-RRHHTDVSFLLDRFTLVGKTQDNK |
|  | SAT1 SAR/9/81 | TTSAGEGAEPVTVDASQHGGNSRGV-HRQHTDVSFLLDRFTLVGKTQNNK |
|  | SAT1 NIG/5/81 | TTSAGEGADVVTVDTAAHGGNQRRT-RRVHTDVAFLLDRFTLVGKTRDNK |
|  | SAT2 KNP/19/89 | TTSAGEGADVVTTDPSTHGGQVVEK-RRMHTDVAFVLDRFTHV-HTNKTT |
|  | SAT2 ZIM/7/83 | TTSSGEGADVVTTDPSTHGGAVTEK-KRVHTDVAFVMDRFTHV-LTNRTA |
|  | SAT2 ERI/12/89 | TTSAGEGADVVTTDPSTHGGNVQEG-RRKHTEVAFLLDRSTHV-HTNKTS |
|  | SAT2 RWA/2/01 | TTSAGEGADVVTTDPTTHGGHPNAA-RRKHTNIAFLLDRSTHV-HTNKTS |
| 50 | SAT1 KNP/196/91 | LTLDLLQTKEKALVGAILRAATYYFSDLEVACVGD-NKWVGWTPNGAPE- |
|  | SAT1 SAR/9/81 | MTLDLLQTKEKALVGAILRAATYYFSDLEVACLGE-NKWVGWTPNGAPE- |
|  | SAT1 NIG/5/81 | MVLDMLKTKEKALVGAILRSATYYFADLEVACVGT-NKWVGWLPNGAPV- |
|  | SAT2 KNP/19/89 | FNVDLMDTKDKTLVGALLRASTYYFCDLEIACVGD-HRRVYWQPNGAPR- |
|  | SAT2 ZIM/7/83 | FAVDLMDTNEKTLVGGLLRAATYYFCDLEIACLGE-HERVWWQPNGAPR- |
|  | SAT2 ERI/12/89 | FVVDLMDTKGKALVGAILRASTYYFCDLEIACVGD-HTRVFWQPNGAPR- |
|  | SAT2 RWA/2/01 | FAVDLMDTKEKALVGAVLRSATYYFCDLEIACVGD-HARVFWQPNGAPR- |
| 100 | SAT1 KNP/196/91 | LAEVGDNPVVFSKGRTTRFALPYTAPHRCLATAYNGDCKYKPTGTAPREN |
|  | SAT1 SAR/9/81 | LEEVGDNPVVFSNRGATRFALPFTAPHRCLATTYNGDCKYKPAGTAPRDN |
|  | SAT1 NIG/5/81 | PREVGDNPVVFSHNGTTRFALPFTAPHRVLATVYNGDCKYKPTNEDTRTN |
|  | SAT2 KNP/19/89 | TTELGDNPMVFSNKGVTRFAVPYTAPHRLLSTVYNGECKYETPVT----A |
|  | SAT2 ZIM/7/83 | TTTLRDNPMVFSHNNVTRFAVPYTAPHRLLSTRYNGECKYTQQST----A |
|  | SAT2 ERI/12/89 | TTQLGDNPMVYAKGGVTRFAIPFTAPHRLLSTVYNGECTYAKTAT----A |
|  | SAT2 RWA/2/01 | TTQLGDNPMVFAHNKVTRFAIPYTAPHRLLSTVYNGECEYTKTVS----A |
| 150 | SAT1 KNP/196/91 | IRGDLATLAARIA-SETH-IPTTFNYGRIYTDTEVDVYVRMKRAELYCPR |
|  | SAT1 SAR/9/81 | IRGDLAVLAQRIA-GETH-IPTTFNYGRIYTEAEVDVYVRMKRAELYCPR |
|  | SAT1 NIG/5/81 | IRGDLATLAARVR-EQSH-IPTTFNYGIILTEAEVDVHVRMKRAELYCPR |
|  | SAT2 KNP/19/89 | IRGDRAVLAAKYS-NIKHTLPSTFNFGHVAADNSVDVYYRMKRAELYCPR |
|  | SAT2 ZIM/7/83 | IRGDRAVLAAKYA-NTKHKLPSTFNFGHVTADKPVDVYYRMKRAAVYCPR |
|  | SAT2 ERI/12/89 | IRGDRAALAAKYA-ASVHTLPQTFNFGFVTVDKPVDVYYRMKRAELYCPR |
|  | SAT2 RWA/2/01 | IRGDRAVLAAKYA-SGKHTLPSTFNFGFVTADKPVDVYYRMKRAELYCPR |
| 200 | SAT1 KNP/196/91 | PVLTHYDHGGRDRYRTAITKPVKQ |
|  | SAT1 SAR/9/81 | PLLTHYDHNGKDRYKTAITKPAKQ |
|  | SAT1 NIG/5/81 | PVLTTYDHALADRYKVSLIAPEKQ |
|  | SAT2 KNP/19/89 | PLLPAYDYASRDRFDAPIGV-EKQ |
|  | SAT2 ZIM/7/83 | PLLPGYDHADRDRFDSPIGV-EKQ |
|  | SAT2 ERI/12/89 | PLLPAYDHASRDRFDAPIGV-ERQ |
|  | SAT2 RWA/2/01 | PFLPAYDHADRDRFDAPIGV-EKQ |

**Reference alignment of the vaccine and infection strains to the study isolates**. The dataset shows the VP2, VP3 and VP1 proteins of the protective strains aligned to the SAT1 and SAT2 isolates used in the study (not shown). The 43 contiguous surface-exposed areas identified by the capsid structural analysis are highlighted. Areas with no amino-acid variability are in grey, and areas with variability are in red for the serotypes for which there is variation.
